# Reduce, Reuse, Recycle for Food Waste: A Second Life for Fresh-Cut Leafy Salad Crops in Animal Diets

**DOI:** 10.3390/ani10061082

**Published:** 2020-06-23

**Authors:** Luciano Pinotti, Michele Manoni, Francesca Fumagalli, Nicoletta Rovere, Alice Luciano, Matteo Ottoboni, Luca Ferrari, Federica Cheli, Olivera Djuragic

**Affiliations:** 1 Department of Health, Animal Science and Food Safety, VESPA, University of Milan, 20134 Milan, Italy; michele.manoni@unimi.it (M.M.); francesca.fumagalli1@unimi.it (F.F.); nicoletta.rovere@unimi.it (N.R.); alice.luciano@unimi.it (A.L.); matteo.ottoboni@unimi.it (M.O.); luca.ferrari2@unimi.it (L.F.); federica.cheli@unimi.it (F.C.); 2 CRC I-WE (Coordinating Research Centre: Innovation for Well-Being and Environment), University of Milan, 20134 Milan, Italy; 3 Institute of Food Technology, University of Novi Sad, Bulevar cara Lazara, 21000 Novi Sad, Serbia; olivera.djuragic@fins.uns.ac.rs

**Keywords:** food waste, animal nutrition, ruminants, vegetable by products, fresh-cut leafy salad crops, former foodstuffs

## Abstract

**Simple Summary:**

There is a historical link between co/by-products and animal feed, however innovative options are now available. Adopting the principles of the circular economy guarantees further progress for the food–feed chain. By-products and biomasses, such as former foodstuffs or plant by-products (PBPs) from the food processing industries, could be recycled as feedstuff for farms. This review focuses on the biomass derived from the processing of vegetables, and in particular on fresh-cut leafy salad crops as potential ruminant feedstuff. The chemical composition of this class of PBPs makes them comparable to other traditional feeds, such as fresh forage, and suggests that they could be considered for ruminant nutrition. Although at a very early stage, the potential of this new biomass seems high. These products can be used to reduce the environmental impact of both the food and livestock sectors.

**Abstract:**

The world’s population is growing rapidly, which means that the environmental impact of food production needs to be reduced and that food should be considered as something precious and not wasted. Moreover, an urgent challenge facing the planet is the competition between the food produced for humans and the feed for animals. There are various solutions such as the use of plant/vegetable by-products (PBPs) and former foodstuffs, which are the co/by-products of processing industries, or the food losses generated by the food production chain for human consumption. This paper reviews the by-co-products derived from the transformation of fresh-cut leafy salad crops. A preliminary nutritional evaluation of these materials is thus proposed. Based on their composition and nutritional features, in some cases similar to fresh forage and grasses, this biomass seems to be a suitable feedstuff for selected farm animals, such as ruminants. In conclusion, although the present data are not exhaustive and further studies are needed to weigh up the possible advantages and disadvantages of these materials, fresh-cut leafy salad crops represent a potential unconventional feed ingredient that could help in exploiting the circular economy in livestock production, thereby improving sustainability.

## 1. Introduction

Food waste is abundant: approximately one third of food produced and intended for human consumption is lost or wasted, which translates into approximately 1.3 billion tons per year on a global level [1]. The disposal of food waste poses a large environmental problem with several implications in terms of the sustainability and profitability of the food system. In order to reduce these negative impacts, the 3R slogan “Reduce, Reuse, Recycle” should be adopted in order to redesign the management of food leftovers and food waste [2,3].

In the European Union, the Waste Framework Directive 2008/98/EC proposed the following waste management hierarchy: prevention, processing for reuse, recycling, energy recovery and disposal [4]. In this scenario, several authors [5,6,7] have suggested that the use of less food-competing foodstuffs in animal diets is a potential strategy for reducing food–feed competition and mitigating the environmental impact of livestock. This approach is particularly pertinent when coupled with other strategies such as improvements in livestock productivity [8,9,10]. Plant by-products include a wide range of secondary residues generated from the industrial processing of plants into commercially valuable products [11]. These products are obtained from agro-industrial processes such as distillery and biofuel production, oilseed processing, fruit and vegetable processing, sugar production, root and tuber processing, and herb, spice and tree processing [12]. These co/by-products are considered safe and are widely accepted as animal feed.

At the food manufacturing level, there are always unintentional and unavoidable food losses that prevent foodstuffs from reaching the human consumption market. Former foodstuff products (FFPs) are a significant example, which have been proposed as animal feed. FFPs are foodstuffs that are manufactured for human consumption, but which are no longer intended for human consumption, despite maintaining important nutritional features [13,14,15,16,17]. Plant co/by-products (PBPs) such as fresh-cut leafy salad crops are potentially another category of former foodstuffs. The present paper addresses the potential of these fresh-cut leafy salad crops (also called salad crops), as a feed ingredient for sustainable ruminant diets.

## 2. From Fresh and Cut Vegetables to Salad Crops: Categorization, Market, Nutritional Facts and Processing

PBPs, are a wide category that includes several types of materials [12]. The main ready-to-eat fresh-cut vegetables and fruits are: arugula and radicchio, parsley, mixed herbs, chard, chicory, puntarelle, rocket in bunches, loose rocket, celery hearts, escarole hearts, courgette flowers, carrots, broccoli, spinach, peeled and sliced potatoes, onion cubes, sliced champignon mushrooms, sliced peaches, mangoes, melons, and oranges [18]. The vegetable products offered to the consumer are based on one or more varieties (mixed salads), which are ready for raw consumption or for cooking (spinach, herbs, vegetable side dishes, legumes). As shown in Table 1, the processing differs considerably according to the type and parts of the vegetable used. In the case of salads, washing, chopping and shredding are the most common processes, while some types of vegetables may also be peeled or cut into slices (slices, rounds or cubes) before being offered to consumers. These latter two treatments are more common for fruits (citrus fruits, pears, apples, pineapples, carrots).

**Figure 1:**
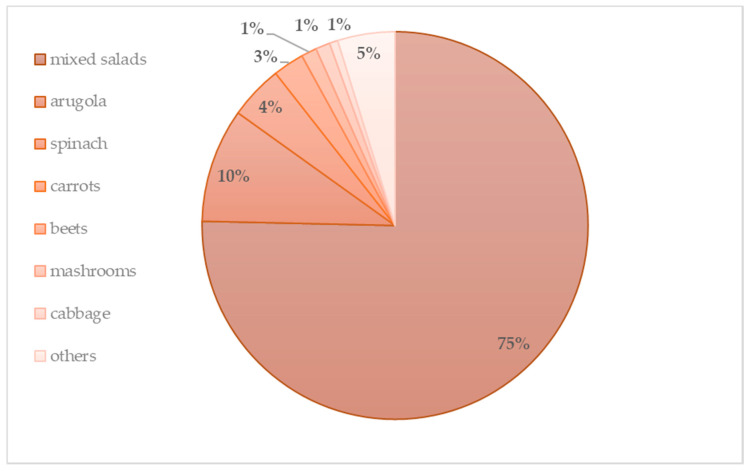
Production of fresh-cut vegetable products in Italy by percentage [19].

The fresh-cut vegetable market, however, is primarily represented by salad crops, including mixed crunchy salads, while single-variety products, such as lettuce, valerian and arugula together represent one third of total sales of fresh-cut fruit and vegetable products. Figure 1 shows the production rate of fresh-cut vegetable and fruit products in Italy.

Salad crops derive from conventional/organic or integrated cultivation systems, and include all those varieties of ready-to-eat fresh vegetables, which during post-harvesting processing are selected, sorter, husked, cut and washed. They are then packed in envelopes or in sealed food trays, and, after passing through the cold chain, are sold on the fruit and vegetable market, ready for raw consumption or for cooking [20].

The salad crop market is increasing. In supermarkets, the space assigned to these products has expanded greatly in order to meet new preparation and presentation styles that are practical both for the consumer and modern distribution. The market share of salad crops is estimated at around 8% of the total fruit and vegetable market in France and Great Britain [18].

**Figure 2:**
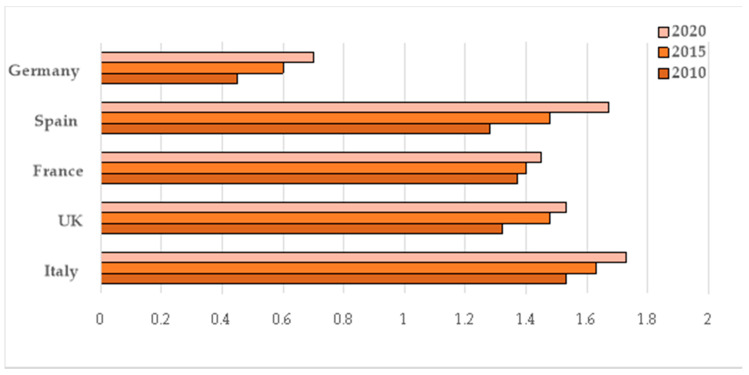
Per capita consumption of salad crops (data expressed as kg/year) [19].

There is a similar situation in Italy, where salad crops now represent approximately 10% of the turnover of fruit and vegetable sales. With about 90,000 tonnes, Italy is ranked as the second largest producer of salad crops in the main European markets, immediately after Great Britain. This scenario is also supported by the per capita consumption of salad crops, which has grown in several European countries in the last 10 years (Figure 2). From this expanding market perspective, the higher the economic and productive importance of salad crop products, the higher the food wastage derived from them [21].

### 2.1. Most Representative Species of Salad Crops and Their Nutritional Facts

Although the generic name of “salad” indicates a group of leafy vegetables consumed mainly as raw material, salads are divided into three botanical families: chicory (which includes radicchio), endives, and lettuce. The following species are the most commonly used in the production of salad crops:

1. Lettuce (*Lactuca sativa*)
2. Arugula (*Eruca sativa*)
3. Endives (*Cicorium sativa*)
4. Valerian (*Valerianella locusta*)

Lettuce (*Lactuca sativa*) is the most important leafy vegetable crop worldwide, and Spain and Italy are the largest producers in Europe [22]. Lettuce is an important component of the modern Western diet as it is consumed in large amounts and contains compounds that are thought to be beneficial to health, particularly flavonoids [23,24,25]. Lettuce has traditionally been sold as a whole head. However, there has been an increase in the proportion of fresh-cut, bagged leaf production because of the increased consumption of convenience foods both in catering and at home. Table 2 shows the gross composition of the most consumed salad crops.

The way fresh-cut salad crops are presented to consumers is another way of classifying the raw materials used in their production: (i) whole-head salad (e.g., iceberg salad), which are vegetables that form a tight cabbage-like head with the leaves branching from a single stalk; (ii) baby salads, also termed baby leaf (e.g., rocket salad), usually harvested at the young leaf stage. The small leaves are supplied intact, which differentiates this product from common cut salads. One of the advantages of baby-leaf salads is that, given the smaller cut surface of the leaves, their color does not fade; various types of baby-leaf salads are available on the market.

### 2.2. Salad Crops: The Production Process

Before being traded and consumed, fresh vegetables undergo a series of technological processes, all strictly based on not compromising the freshness and naturalness of these products. Conserving the organoleptic properties depends on the processing procedures, preservation techniques, and the time required for the product to reach the dealer, beginning at the processing plant. The preservation of salad crops is based on the combined action of different treatments, which are all designed to prevent bacterial contamination and delay the appearance of alterations and spoilage.

When processing a whole-head salad, cutting operations are required. Cutting damages the plant tissues, which consequently reduces their quality during storage. Moreover, in the case of whole-head salads, the percentage of usable product is significantly lower (due to the preliminary removal of the external leaves and core) than in baby salad processing, for which the whole leaf is harvested and processed [26]. Whole-head salad processing is thus responsible for a huge amount of waste. The total wasted salad can be calculated as the sum of the waste generated during preliminary cleaning, the three washing stages, and the waste generated at the optical selector step. Data indicate that up to 41% of salad is wasted during typical fresh-cut iceberg salad processing because of the removal of the external leaves and core, accounting for nearly all the total waste production [27].

**Figure 3:**
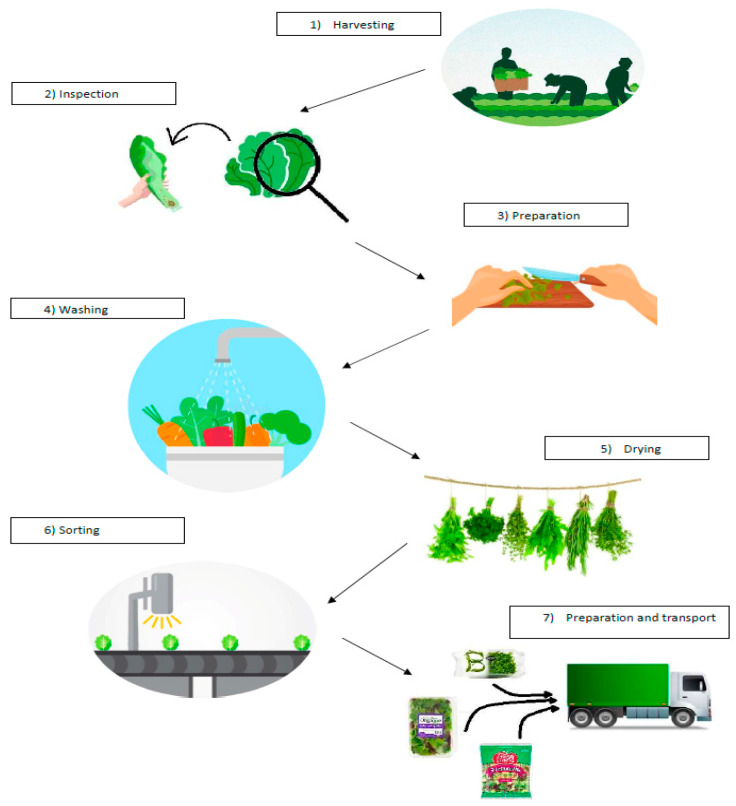
Salad crop production process.

A generic example of the steps that salad crops undergo before being marketed is represented in Figure 3. It is clear that waste production occurs at several levels of the production chain.

The main salad crop production steps can be summarized as follows: selection (choice of variety); cleaning and washing (water moves through mechanical or air agitators); cutting (when needed); re-washing and drying (in order to guarantee a limited microbiological load); packaging (needed to keep the characteristics of freshness for the shelf life of the product); labelling; retailing; and transportation.

## 3. Salad Crops as Animal Feed Ingredients

A feasibility study was carried out at the University of Milan in conjunction with a salad processing plant, in order to evaluate the chemical and nutritional properties of different kinds of salad crops as animal feed sources. Several samples of salad crop leftovers were collected in Autumn 2019. Samples were analyzed in relation to dry matter (DM), crude protein (CP), crude oils and fats (EE), ash, neutral detergent fiber (NDF), acid detergent fiber (ADF), and acid detergent lignin (ADL). Specifically, the DM, EE, CP and ash analyses were performed in compliance with Commission Regulation N° 152/2009 [28]. Neutral detergent fiber, ADF and ADL analyses were performed in accordance with the methods 2002.04 and 973.18 for NDF and ADF-ADL, respectively [29]. The energy content was estimated using the equations proposed by Weiss [30], and data reported by CRPA [31].

### 3.1. Comparison of Macronutrients

Table 3 compares the nutritional value of fresh forage (grasses and legumes) and salad crops. The results show that these materials are similar to fresh forage or pasture used in conventional ruminant feed. One of the main differences is the water content: the dry matter (DM) concentration ranges from 6% in salad crops to 23% or 25% in forage [31]. This indicates that salad crops are extremely wet, which is usually related to the age of the grass. Salads contain a lot of water, which provides high palatability and high bulkiness. Bulkiness is an important feature in terms of their mechanical action on rumen walls, and it can be adapted according to the biomass available from the salad crop processing plant. For example, a salad variety with a fast fiber clearance and degradation rate could reduce its residence time in the rumen, ensuring more space for extra material to be ingested, and this would have a positive impact on increasing DMI, especially in early lactation in dairy cows [32].

However, the main components of these materials are crude proteins (CP) and fibers (NDF, ADF, etc.). The crude protein content is very high and comparable to lucerne grass. The overall mean protein content in salads is about 21% DM. This value is in line with Bakshi et al. [33], who have reported that vegetable waste has about 20% CP, high moisture and high palatability. These data, however, should be interpreted with caution since different protein fractions were not reported. Usually young grasses are characterized by a high proportion of soluble nitrogen: in most feedstuffs, a large fraction of soluble nitrogen is in the form of non-protein nitrogen.

As seen from the present analyses (Table 3), the protein content in salad crops is high, but it is not clear whether this content derives from proteins or from non-protein nitrogen. These foodstuffs contain high concentrations of soluble nitrogen, which, if not properly balanced, can have a negative effect on the diet. In fact, high soluble proteins, not accompanied with an adequate carbohydrate intake, can cause lameness or meteorism in animals. At an early stage of development, both grass and salads are rich in water and proteins, due to the intense metabolic activity of the tissues [34].

Nevertheless, it would also be interesting to know the amino acidic profile of salads and the degradability and digestibility rates of proteins, and whether there are bypass proteins. In terms of amino acids content, it has been reported [35] that glutamate and/or glutamine are the predominant amino acids in the leaves of all salad crops species. However, how these amino acids are distributed among the different proteins fractions is unknown, even though a high incidence of soluble protein is expected. This fraction has several dietetic implications that need to be addressed. One recent study [36] reported high rates of in vitro digestibility and rumen fermentation kinetics of selected vegetables. Specifically, de Evan and coworkers [36] reported that the rumen degradability of vegetable proteins at 12 h of in situ incubation was greater than 91.5% for all tested materials, while the in vitro intestinal digestibility of proteins ranged from 61.4 to 90.2%. Thus, when exposed to an artificial rumen environment, proteins and sugars from the tested vegetables were rapidly and extensively fermented. Furthermore, the same materials, when mixed with conventional ingredients, also had a reduced in vitro methane/total volatile fatty acid ratio.

As seen from the same analysis (data reported in Table 3), salad crops are also rich in fiber, as demonstrated by the NDF content that reached 36% DM, i.e., not far from the value reported in legume forage (40% DM), but lower than forage grasses (58.2% DM). The ADF content is about 24% DM, however this is lower than fresh forage (legumes and grasses) probably due to the early growth stage of these baby leaves.

These results indicate a different potential in the ruminal degradability of salad crop fiber, which introduces a further issue for salad crops, i.e., digestibility. Digestibility is influenced by the relationship between the leaves/stems: in very young herbs, as in baby leaf salad crops, the stems appear more digestible than the leaves, but later in the phenological stage, the digestibility of the leaf fraction decreases very slowly, while that of the stems decreases rapidly [34]. These features suggest that salad crops are a potential source of highly degradable fiber. A comparison with sugar beet pulp (excellent fiber source) would thus be useful here [36]. For instance, salad crops have a lower NDF content (36.0% DM) than beet pulp (48% DM). In addition, while the ADF content is the same in salad crops and sugar beet pulp (on average 23–24% on a DM basis), the ADL fraction is higher in salad crops (around 7% on a DM basis) than in sugar beet pulp (less than 3% on a DM basis). An important focus should be the evaluation of the physically active NDF. All these data are necessary to evaluate the possible levels of inclusion and the possible changes in rumen microflora and related fermentations.

Further, as reported in Table 3, the ash content is very high in salad crops (18.5% DM), even twice as much as in common forage (9.5% DM grasses, 9.7% DM legumes). The high value of ash content could correspond to contamination during harvesting by soil remaining on the leaves and other vegetable parts. Usually, the forage ash content comes from both internal and external sources. Internal sources include minerals that accumulate in the leaves and stems of forage plants, but this is probably not the case for young materials such as salad crops. External sources include soil and sand that are deposited on the surface of the forage. An average internal ash content for grasses, as reported in Table 3, is around 9% DM. Values above this represent external sources and are negatively associated with forage quality and animal performance because the other nutrients are replaced by ash. Although a high ash content is an intrinsic feature of this type of biomass, salad crops are a leftover and thus they contain a higher percentage of soils and contaminated leaves than salads that pass through all the processing steps and reach the market, because the majority of salad crops are subject to the cleaning and washing involved in the production chain.

Although the results reported in Table 3 should be interpreted with caution since they are case sensitive, i.e., they thus represent a few examples of the different ex-foods that are potentially available for the feed stock used for feeding ruminants, the key features in this respect are the nitrogen and fiber contents. Some varieties from the same botanical families of salad crops have already been proposed as ingredients in ruminant diets. For instance, Marino and coworkers [37] investigated the potential nutritional value of vegetables and fruits recovered from a supermarket. Even though they observed a similar protein content for leafy vegetables, the NDF and EE contents were different. In their study [37], the NDF content in leafy vegetables was about 40%, slightly higher than in our study, while the fat content was almost twice the amount we found. Both these aspects also affected the estimated energy content, which, as expected, reached about 8 MJ/kg^−1^ DM [37].

García-Rodríguez and collaborators [38] recently assessed the nutritive value of 26 agro-industrial by-products (sugar beet, asparagus, different citrus pulps, lettuce, etc.) in terms of chemical composition, in vitro digestibility, and rumen fermentation kinetics. The results showed differences in chemical composition, in vitro digestibility and fermentation kinetics, even in the same by-product processed in different ways (e.g., dehydrated/ensiled sugar beet pulp, tops and leaves). Taken together, these results indicate that composition variability seems to be one of the main limitations in defining a possible scale-up for the use of these biomasses [38]. The salad crops here analyzed, however, have shown some similarities with brassicas forages: both contain high levels of easily fermentable carbohydrates, which can improve DM digestibility and ruminal fermentation. Furthermore, these compositional features have been associated with a reduction in the acetate-to-propionate ratio and energy losses in the rumen (mainly methane emissions), which in turn improved feed efficiency [39]. This is in line with other studies [40], in which the progressive inclusion of fresh grass in lactating dairy cows’ diets linearly increased milk yield (+0.21 kg/d per 10% proportion of fresh grass in the diet). In terms of composition, fat yield was unchanged, while fat content was slightly reduced. A side effect was also on milk fat globule size, which was decreased when the proportion of grass reached 30% in the diet. This latter also affects the technological quality of milk fat and of the resulting butter. Even though the nutrient fractions responsible of these effects are unknown, a combination of fiber and protein portions in the rumen seems to be the best option. Intuitively, there are no studies on the use of salad crops in dairy cows’ diets and their effects on milk production and product quality, but their features are promising.

### 3.2. Micronutrient Content in Vegetables

Salad crops are a major source of micronutrients, such as vitamins C, B complex (thiamin, riboflavin, B6, niacin, folate), A, E, as well as minerals and polyphenols, carotenoids, and glucosinolates [27]. The main bioactive compounds in salad crops are summarized in Table 4.

Despite this long list (Table 4), there are few studies in the literature regarding possible effects of this novel ingredient on animal performance, yet it is likely that they can improve some aspects of animal performance, such as productivity and reproductive parameters, and also reduce methane and nitric atmosphere emissions [33,41]. As proved in vitro by Oskoueian et al. [41], selected bioactive compounds such as naringin and quercetin (both flavonoids), at the concentration of 4.5% of the substrate (dry matter basis), were able to suppress methane production without any negative effect on rumen microbial fermentation and total populations of protozoa. Accordingly, methanogens were significantly suppressed by adding these compounds.

The presence of some bioactive compounds, such as flavonoids, could improve not only the rumen functions but potentially also animal wellbeing and product quality. Fresh herbs could modify the acidic composition of lipids in milk and thus improve the quality of ruminant products. These qualitative aspects can also concern meat production, with enhancements to the intramuscular lipid fatty acids profile, reducing the fraction of saturated fat. However, there is little evidence on these aspects and further investigation in controlled studies is needed to define the real potential of salad crops.

### 3.3. Nitrate Monitoring

Under certain soil and environment conditions, plants can accumulate nitrates. Virtually all plants have the capability of accumulating nitrates. The amount of nitrate in plant tissues is affected by several factors like: plant species, stage of maturity, part of the plant. Above these “plant factors”, other things/practices can affect the uptake and accumulation of nitrate by plants, namely nitrogen fertilization, herbicide application, drought, cloudy or cold weather, etc. However, nitrate concentrations are usually higher in young plants and decrease as plants mature. It is known that several vegetables, including salad varieties, have the potential to accumulate nitrate under specific growing intensive conditions [42]. In the rumen, ingested nitrate is broken down to nitrite and then undergoes further degradation to ammonia, which is used to form microbial proteins. The reduction of nitrate to nitrite occurs much more rapidly in the rumen than the reduction of nitrite to ammonia. Consequently, when ruminants consume plants high in nitrate, excess nitrite formed in the rumen enters the bloodstream where it converts blood hemoglobin to methemoglobin, which, when excessive, may induce nitrate poisoning. Plants containing more than 1% nitrate (10,000 ppm) have to be managed with caution, and nitrate consumption in amounts of as little as 0.05% of the animal’s weight can be dangerous. Forages containing more than 1% nitrate can be fed if diluted with nitrate-free plant material [42]. However, recent surveys [43] conducted on lettuce, rocket, spinach and other leafy green vegetables, have evidenced a nitrate concentration between 2800 to 4130 mg/kg, which is very far away from the risk limits.

## 4. Salad Crops: Future Perspective as a Feed for Ruminants

The farm management of salad crops entails risks associated with the high-water content and possible undesirable fermentations. Firstly, the ways in which salad crops can be fed to animals should be evaluated. Salad crops can be used fresh and mixed in the diet with total mixed ration (TMR), dried or in silage. Drying and ensiling are attractive means to preserve vegetable waste and by-products, although dehydration seems to be the least effective solution in terms of energy inputs and cost. In general, convectional drying processes, commonly used for forages, such as open solar drying, have some drawbacks in terms of quality, capacity, accuracy and process efficiency. On the other hand, fossil-fuelled dependent drying systems presented other drawbacks, indeed such technologies are often uneconomical and unsustainable from an environmental point of view. However, as reported elsewhere, when food leftovers or waste are considered as animal feed, their dry matter content is a key issue. In this respect, the most exhaustive example comes from Vandermeersch et al.’s [44] study, which has made a direct comparison between food leftover processing and biogas production of ‘bread waste’. In that study, it was pointed out that valorizing food waste to animal feed seems to be the better option, especially for those fractions of food waste with low water content (such as bread waste). Thus, since salad crops are very wet materials, their management imposes that they be considered “as is” for conversion into animal feed ingredients. These aspects are also linked to the energy, water and food (EWF) nexus, which refers to the interdependencies that inherently exist between these resources.

An alternative solution is ensiling these materials. Silage includes multiple harvest, transport, and storage operations, while preservation is guaranteed by fermentation. In order to guarantee high quality silage, by maintaining economic profitability, all of these need to be coordinated, and the number of equipment components needs to be adjusted according to the processing capacity. In this respect, however, it is known that forages that have excess moisture (>70%) can get unintended fermentations (e.g., clostridial fermentation). This aspect (DM content) is the most important for fresh salad crops that are extremely rich in water. Wilting high-moisture forage to at least 35% DM is a good practice that reduces dangerous fermentations. Wilting usually results in good silage, particularly when sugar concentration is low and buffering capacity against pH decline is high [45]. However, the use of ensiling in salad crop fields has rarely been studied in the literature, which limits an adequate evaluation of this process. Consequently, using fresh salad crops in the diet with a TMR is the most common and easiest way to incorporate them into animal feed. This, however, may be affected by the distance between the producing plant and the potential users: salad crops should be used/fed fresh in the surrounding areas of the vegetable processing plants [33].

More than a quarter of all salad crops are estimated to be processed into prepackage salads. Interest in ready-to-eat salads is still increasing worldwide and has an enormous potential for further growth. Italy has the highest per capita consumption of fresh-cut salads in Europe and produced 110,000 tons of fresh-cut vegetables in 2015, with a value near to € 750 million [46]. The increase in the fresh-cut vegetables market implies the potential production of high amounts of food leftovers that could be converted to animal feed ingredients [21]. The salad crops market combines the advantages of ready-to-eat foodstuffs, convenience and innovation, with healthy eating. The innovation represented by this sector involves the technologies adopted in growing, processing, and marketing. Agronomists, microbiologists, chemists and food engineers are providing new solutions to enhance quality and safety attributes.

Like all fresh-cut products, salad crops require substantial capital investment in plants and machinery. For this reason, in Europe, dairy processing plants often also specialize in ready-to-eat salads, since both products require daily delivery [46]. This link between the two sectors should be exploited in the development of an integrated system in which the surplus of salad production returns to the food chain by being introduced into the diet of dairy cattle. An integrated system of this kind could help make the use of salad crops in the diets of farm animals economically sustainable. Indeed, the main problem regarding salad surpluses is the high-water content, which makes any handling and processing economically uncompetitive. By contrast, the re-use of these biomasses (salad crops leftovers) in ruminant diets is a sustainable solution that saves not only nutrients, but also a huge amount of water, from waste.

In order to correctly manage salad crops and consider them as a possible animal feed resource for a sustainable ruminant diet, it is essential to investigate how they are processed. Companies need to ensure that all the processing of fresh-cut vegetables (including salad crops), from the selection of the product to the transport and sale, takes place within 24 h. Usually, the process takes 6–7 h for the product to pass from harvesting to the completion of the processing phase, and the remaining 17–18 h are needed to reach the retailers. The leftover biomass is generated in the processing plant and is stored in transport semitrailers until their departure to the final destination. In this scenario, pH levels could be determined in order to assess the freshness of the leftovers: according to our analysis, the most appropriate value is around 6 or neutral (Pinotti, unpublished results). In addition, supplementation with salad crops in the diet should be introduced gradually in order to evaluate the effects on animals.

From a nutritional point of view, there may be differences depending not only on the agronomic/cultivation factors [35], but also on the steps performed after harvesting and during processing. A key example is cutting, which subjects the salads to more alterations, due to the stimulation of ethylene production, which increases respiration and senescence, and exposure of the cutting surface to microbial enzymes and potential spoilage. Furthermore, as highlighted by the high value of ash, these products may have large traces of soil derived from field harvesting. A decrease in nutritional values is also expected when plant tissues are wounded, and in vivo data indicate that fresh-cut lettuce contains fewer antioxidants than the fresh product [47]. However, little information is available concerning the effects on nutritional components, particularly antioxidant constituents, in fresh-cut products during handling, storage and senescence.

In summary, salad crops are potentially suitable ingredients for the feed stock supply for feeding ruminants, as demonstrated by their nutritional features as well as the high biomass content derived from the processing plants.

## 5. Conclusions

This article summarizes the features and potential of salad crops as new ingredients in ruminant diets. These types of feed are an example of the application of the principles of the circular economy and, given their nutritional value, are a potential alternative to conventional feeds such as forage used within the conventional ruminant diet. In fact, in terms of animal nutrition, these feeds have many benefits—especially for ruminants—because of their high fiber and protein contents. Their nutritional features, however, can also be considered for supplements and feed specialty formulation. Fresh salad crops, indeed, are recognised to be important for human diet due to their abundance in micronutrients like minerals (e.g., potassium, calcium and phosphorus) as well as vitamins (mainly A, C and E). Salads also contain bioactive phytochemicals such as carotenoids, polyphenols, glucosinolates and CLA. The main classes of polyphenols are caffeic acid derivatives, flavonols and anthocyanins, which play an important role as antioxidants [27,48]. The processing steps of salad crops (reviewed in Paragraph 2.2) influence the micronutrient content of salads and, although some studies have been carried out [26,49], further research is needed to elucidate the variation of micronutrients content in fresh-cut products and their potential as animal feed. These features, in combination with their palatability, can be exploited to prepare new formulations that can be used as supplements in specific phases (e.g., early lactation, early dry period).

In addition, the high content of available biomass, due to the increasing market of fresh-cut products, ensures that these leftovers can be continuously implemented in the feed industry. Key to their successful use is how they are managed, from the waste origin to administration on the farm. Their nutritional characteristics need to be better understood in order to be able to use them correctly and to prevent the risks associated with their use.

We believe that future work should first investigate how salad crops are processed as animal feed. The focus should then be on the nutritional and functional role of specific nutrients, which could positively affect the animal’s performance, but also affect their digestibility, firstly in terms of proteins (it is not clear whether the nitrogen content derives from proteins or from non-protein nitrogen) and then the ash content (a high ash content is an intrinsic feature of this type of biomass, due to soil contamination).

## Figures and Tables

**Table 1:** Fresh-cut vegetable products: parts of the plants and processes performed on the products.

Fresh-Cut Vegetables	Parts of the Plant Used	Processes Performed on the Product
Lettuce	Leaf	Cleaned, chopped, shredded
Spinach	Leafy greens	Washed and trimmed
Broccoli & cauliflower	Flower	-
Cabbage	Leaf and flower	Shredded
Carrots	Roots	Shredded
Onions	Bulb	Whole peeled, sliced, diced
Potatoes	Roots	Peeled, sliced, diced
Garlic	Bulb	Fresh peeled, sliced
Tomato	Fruit	Sliced

**Table 2:** Chemical composition of most consumed salad crops (g/100 g of fresh material).

Salad Crops	Water Content	Fiber	Protein	Carbohydrate	Fat	Ash	MJ/100 g
Lettuce	94.61	2.1	1.23	3.29	0.3	0.58	17
Arugula	91.71	1.6	2.58	3.65	0.66	1.4	25
Endives	93.79	3.1	1.25	3.35	0.2	1.41	17
Valerian	92.8	1	2	3.6	0.4	1.2	21

**Table 3:** Comparison between nutritional values of fresh forage and salad crops.

Item ^1^	Salad Crops	Fresh Forage ** (Grasses)	Fresh Forage ** (Legumes)
Dry matter (DM), %	5.80	25.0	23
CP, % DM	21.2	8.80	18.9
EE, % DM	2.85	1.55	2.8
NDF, % DM	36.0	58.3	40
ADF, % DM	23.8	39.0	31
ADL, % DM	7.40	5.39	7
Ash, % DM	18.5	9.94	9.7
NEL, MJ/kg *	3.70	3.90	4.18

^1^ crude protein (CP), crude oils and fats (EE), ash, neutral detergent fiber (NDF), acid detergent fiber (ADF), and acid detergent lignin (ADL); * The Net Energy Lactation (NEL) was estimated using the equation from [30]; ** [31].

**Table 4:** Major bioactive compounds found in vegetables [27].

Vegetables Classification	Bioactive Compounds	Function in Human
Cabbage (*Brassicaceae*)	Glucosinolates (GLS): Isothiocyanates (ITC), sulforaphane	Fungicide, bactericide, nematocide chemopreventive, cardioprotective, anti-inflammatory
Carrots, Lettuce	Beta-carotene	Antioxidant
Lettuce, Spinach	Flavonoids	Antioxidants and anti-inflammatory
Lettuce	Genistein, daidzein, anthocyanins, lycopene	Hypoglycemic
Endive	Bitter substances	Bile-production stimulants, pre-probiotics, expectorants
Valerian	Soluble fibers	Anti-inflammatory Tonic
Spinach	Saponins	Diuretic
